# Does religious faith contribute to the preservation of personal value system in patients with schizophrenia? an empirical research

**DOI:** 10.3389/fpsyt.2025.1553990

**Published:** 2025-04-07

**Authors:** Alexey M. Dvoinin, Grigoriy I. Kopeyko, Olga A. Borisova, Ekaterina V. Gedevani

**Affiliations:** ^1^ School of Psychology, HSE University, Moscow, Russia; ^2^ Department of Transcultural Psychiatry, Mental Health Research Center, Moscow, Russia

**Keywords:** personal values, personal value system, religious faith, religiosity, schizophrenia, mental illness, Orthodox Christian, religious patients

## Abstract

**Introduction:**

As demonstrated in previous research and clinical observations, the personal value system is subject to disintegration as a consequence of schizophrenia. Patients with schizophrenia are sometimes religious and use religious coping mechanisms. A number of studies emphasize the benefits of positive religious coping as a part of clinical treatment for patients with schizophrenia and schizoaffective disorder. However, the contribution of these patients’ religious faith to the structure and composition of their personal value systems remains unexplored. The present study attempts to answer this question.

**Method:**

The factorial design (2x2) included two conditionally independent variables: mental illness (absent/present) and religious faith (absent/present). We sampled four groups (N = 65) for the study: mentally ill believers of Orthodox Christian faith, mentally ill non-believers, healthy believers, and healthy non-believers. We analyzed the structure and composition of the participants’ personal values employing the following tools underpinned by G. Kelly’s personal construct theory: the triad method, Hinkle’s laddering and repertory grid methods. Correlation and factor analyses were then conducted within each group to find the relationships between the personal values identified. Subsequently, we compared the personal value systems of each group with each other.

**Results:**

The outcomes of the study reveal that personal values of healthy non-believers are less differentiated than those of healthy Orthodox believers and can be divided into two clusters of meta-values: spiritual and material. Mental illness in non-religious individuals is likely to contribute to disintegration of their personal value systems. Healthy believers have distinctly differentiated and hierarchical personal value systems, while mentally ill believers retain both the general hierarchy and key structures of their personal value systems.

**Discussion:**

The relative stability of the personal value systems of mentally ill believers is explained by their attitude toward illness as a form of trial, which is integrated within the framework of their religious worldview grounded in the Orthodox Christian doctrine. In this way, illness is not regarded as a hindrance to achieving life goals and personal meanings inspired by religion.

## 1 Introduction

As demonstrated in previous research and clinical observations, one of the adverse consequences of schizophrenia’s impact on personality is the disintegration and transformation of personal values (20, 33, 60). As the disease progresses, the range of values narrows, and the patient experiences disintegration in various forms, including feelings of helplessness, a perception of a meaningless existence, and a loss of control over life.

An integrated system of personal values has been demonstrated to be associated with mental health. Values are regarded as a central component of the self (49). Therefore, building up the personal value system in patients grappling with mental illness is often considered a key component of psychotherapy and rehabilitation practices (24, 28, 43).

Numerous studies have produced compelling evidence for higher effectiveness of therapeutic efforts in patients holding religious beliefs or spiritual aspirations (18, 50, 62). Patients with schizophrenia are sometimes religious and use religious coping mechanisms (26, 27, 32, 45). However, the contribution of these patients’ religious faith to the structure and composition of their personal value systems remains unexplored.

The article is based on supplemented and revised research data, partially presented earlier in a Russian-language version (4).

### 1.1 Personal values and personal value system: theoretical considerations

In the most general sense, by ‘*personal values*’ we mean cognitive representations of motives as broad, conscious life goals to be pursued (49). These mental structures are distinguished by four specific features.

First, personal values are defined as guiding principles that inform behavior, attitudes, and motivations beyond the context of a particular situation. In this way, they impart a sense of direction to an individual’s life, ensuring stability in diverse contexts and over time. This attribute differentiates values from motives and attitudes, which are more fleeting and situational in nature.

Second, unlike personality traits, values are desirable goals that a person deems good and worthy. They are inherent in activity and are meant to be realized (49). Values enable us to prioritize decisions and choose behaviors consciously.

Third, values are socially conditioned and socially desirable, promoting cooperation and constructive interactions with other people. They serve as the foundation for the cohesion of social groups.

Fourth, personal values are involved in the meaning-making process. Some researchers define values in terms of meaning, as a person’s conscious and accepted general meanings of his or her life (6). Individuals use values to assess the significance of events, phenomena, people and life choices. In other words, values can serve as personal constructs, as Kelly (29) argues.

In the present study, it is important to trace the contribution of the religious faith of patients with schizophrenia to the *systemic organization* of personal values. To this end, we use the concept of ‘*personal value system*’ (PVS), which is defined as a set of personal values that are interrelated and function as a whole.

This concept underscores specific structural and organizational components of personal values and assumes that:

1. a specific personal value may be associated with more, fewer, or no other values;
2. the associations between personal values may be positive or negative, strong or weak;
3. the personal value system may be more integrated (large number of associations) or differentiated (small number of associations). It is possible to identify the most associated values (meta-values); and
4. the personal value system is typically characterized by a hierarchical structure, wherein more significant values are distinguished from less significant values. The most important values function as system-forming elements, also known as the ‘core’ of the system.

There is now ample evidence of differences in personal values between believers and non-believers. According to Roccas and Elster, differences in behavioral patterns between believers and non-believers are based on value differences (48). This is not surprising since any religion imparts to its adherents a system of values and norms designed to regulate their behavior.

Studies conducted by Roccas and Elster (48), Saroglou et al. (51), Schwadel and Hardy (54), and Schwartz and Huismans (56) have indicated a positive correlation between religiosity and the values of Tradition, Benevolence, and Conservation. Conversely, a negative correlation has been observed between religiosity and the values of Openness to Change, Achievement, and Self-Enhancement (in terms of Schwartz’s value theory). The relationship between the value of Conformity and religiosity varies significantly, depending on the characteristics of the sample studied, the specific religion and the component of religiosity (e.g., religious affiliation, religious practices, religious beliefs, etc.).

The PVSs of believers are specific – religious and non-religious values can be integrated or be relatively autonomous, i.e., they can function as two distinct subsystems and regulate behavior in different ways (14).

### 1.2 Personal value system in patients with schizophrenia

Some personal values (Conformity, Benevolence, Hedonism, Self-Direction) can serve as predictors of mental health if they are mediated by social support or resilience (38). However, this pattern has cross-cultural specificity: it is more apparent in traditional societies than in post-industrial ones (38). In certain instances, an abnormal built-up of one’s PVS can either predispose a person to a serious mental condition or be its main symptom, a consequence of how an illness which was already present transforms one’s attitudes toward the external world (25). In cases of severe mental disorders, such as schizophrenia, the PVS narrows into a handful of values, resulting in a dramatic decrease in motivation to act or seek meaning in one’s actions (63).

Studies on the personal values of patients with schizophrenia have produced ambiguous results regarding the structure of their PVSs. Some findings suggest that while healthy people hold the same set of core values (e.g., *love*, *family*, *trust*, *friendship*) throughout their life, mentally ill individuals tend to experience a complete loss of their value systems within approximately 40 years following the onset of their illness. In individuals diagnosed with mental illness, values no longer maintain their socio-cultural significance; having previously been abstractions, they become embodied in some physical form (37). Affected individuals tend to assign value to interpersonal relationships, while healthy individuals prioritize personal values (e.g., *creativity*, *freedom*, *recreation*, etc.). According to estimates made by Leontyeva and Golovina, 25% of individuals diagnosed with schizophrenia reject the values and norms of their society, and consequently fail to adapt (36). Patients who have suffered a major schizophrenic episode score low on the scale of how meaningful their life seems to them (43).

Considering values as action-guiding attitudes, Stanghellini & Ballerini concluded that the schizophrenic value system reflects a crisis of common sense and is characterized by *antagonomia* and *idionomia* (59). Antagonomia involves choosing an eccentric stance in opposition to conventional value beliefs, while idionomia signifies a sense of uniqueness and exclusivity in relation to others and common sense. The authors argue that this sense of radical exceptionalism can be perceived as a ‘gift’ and is often associated with an eschatological mission or a calling to a higher, metaphysical understanding of the world (59). As we suggest, this aspiration toward eschatology and metaphysics can be infused with religious or spiritual values.

Despite the abundance of data underscoring discrepancies in PVSs between individuals with and without mental illness, a number of studies offer contradictory insights. A notable finding is the tendency of both mentally ill and healthy respondents to prioritize *love*, *family*, *health*, *friendship*, *freedom*, and *material well-being* as their core values (35). This observation suggests that, in terms of spiritual and anthropometric features (7), patients with schizophrenia are not distinctly different from their healthy peers. Vityunina (60) reported no significant disparities in life orientations between individuals with schizophrenia and healthy individuals, as all metrics indicating the meaningfulness of their lives remained within normal parameters. However, the author noted that PVSs of patients with schizophrenia are limited and distorted (60).

### 1.3 Religiosity and spirituality in patients with schizophrenia

Previous studies suggest that religious beliefs and spirituality are of high importance for people with psychiatric disorders, both in terms of quality of life and life strategies (5, 12, 41), as they provide a rationale for their condition and help choose a means of coping (47).

Studies of patients with schizophrenia, borderline personality disorder, bipolar disorder and anorexia indicate that for 26% of affected individuals, their religious beliefs and spiritual practices play a pivotal role in ensuring the quality of their life by making it more meaningful to them. Spirituality has been found to be strongly associated with enhanced social functioning, higher self-esteem and fewer negative symptoms; it affirms concepts of moral universalism, Christian humility and helpfulness (24, 25). Furthermore, faith in God has been shown to help patients feel less stigmatized by their condition (21, 32). This is due to the ability to incorporate their condition into a religious narrative and redefine it in religious terms.

A number of studies highlight the benefits of positive religious coping as a part of clinical treatment for patients with schizophrenia and schizoaffective disorder (20, 32, 33, 41, 57). Patients who believe in God and have the opportunity to engage in religious rituals demonstrate enhanced adaptive functioning, characterized by superior self-control, stronger emotional attachment to others, greater emotional comfort, and a heightened sense of purpose in spiritual practices and life in general (45).

According to Mohr et al., who studied positive and negative religious coping mechanisms in patients with schizophrenia and schizoaffective disorder, positive religious coping has been shown to be a predictor of a reduction in negative symptoms, enhanced social functioning, and an improvement in quality of life (41). However, the frequency of religious rituals in a religious community or communal support does not appear to have any effect on the outcome of treatment. Another study found no significant differences in religious coping mechanisms between patients diagnosed with schizophrenia and those diagnosed with schizoaffective disorder (57).

The positive effect of religiosity/spirituality on the lives and rehabilitation of patients diagnosed with schizophrenia has been observed in a variety of cultural contexts. Researchers have noted similar trends in Christian-oriented patients from Western countries (Switzerland, Canada and the USA). Helpful religion has been associated with improved social, clinical and psychological status (39). A similar trend has been observed in Russian Orthodox patients (20) and ultra-Orthodox Jews (57). Christian patients with schizophrenia from the Hungarian sample also showed an increase in religious and spiritual aspirations, which was predicted by more severe self-disorder, perceptual disturbance, and positive clinical symptoms (30). Religious activities such as ‘salat’ and ‘dhikr’ have a positive effect on the physical and mental health of Muslim patients in Indonesia (27). A study of Shona people in Zimbabwe revealed that religious faith and religious affiliation offered a network of support for patients with schizophrenia, fostering hope, resilience, a sense of self-worth, and an enhancement in quality of life. Furthermore, cultural and religious beliefs have been found to influence the perceived causes of schizophrenia, explanations of symptoms, and help-seeking behavior (9).

However, we would like to note that the prevailing tendency of religious coping to positively influence the treatment process of patients with schizophrenia needs to be clarified, for two major reasons.

First, this tendency appears to be associated more with improvements in patients’ psychosocial functioning than with reductions in the clinical symptoms of the disorder. In some studies, the association of religious coping with the severity of mental disorder is either small or statistically insignificant (see, e.g., 10, 26). These findings emerge from the attempt to identify linear correlations between patients’ religiosity/spirituality, the course of their illness and their psychosocial well-being. It is possible that these associations may be nonlinear, potentially influenced by mediating or moderating factors.

Second, researchers have identified a less beneficial relationship between the religiosity/spirituality of patients with schizophrenia, on the one hand, and their clinical and psychosocial statuses, on the other. For instance, Amadi et al. discovered that non-organizational religious activity and internal religiosity are negatively correlated with psychopathological symptoms (1). Other studies have demonstrated that spirituality can also become a source of suffering (3, 23, 39, 40), while strong religious views can exacerbate a mental disorder and conflict with psychiatric treatment (21, 23). However, such negative effects are much less common than positive ones.

This ambiguity can be explained by the differences in the samples studied, including various confessional, ethno-cultural, gender, age, and socio-demographic characteristics of patients, as well as different stages of disease and treatment strategies. Additionally, the research design and methods, such as qualitative or quantitative approaches, contribute to the observed variations. It is important to note that the studies also measure very different components of religiosity.

An alternative explanation also merits consideration. We propose that the underlying cause of this ambiguity may be attributed to a combination of two manifestations of religiosity observed in patients:

1. when religiosity is a ‘symptom’ of a psychopathological syndrome (manifested as religious delusions, hallucinations, etc.);
2. when religiosity is not a symptom of schizophrenia, but is the result of a patient’s search for a resource for coping with the illness.

While the presence of religious delusions and hallucinations is common among individuals diagnosed with schizophrenia, these symptoms are heavily influenced by the cultural context in which the individual is raised. This can complicate the distinction between culturally sanctioned spiritual practices and those that are considered psychotic (31).

This distinction is significant, because when patients’ religiosity is pathological in nature, there is almost no difference in the response to treatment between religious and non-religious patients (30, 58). Conversely, when the religiosity of patients with schizophrenia is conditionally healthy, it can serve as a significant rehabilitation resource if the specifics of their PVSs are taken into account (20, 33).

In any case, it is clear that the religious beliefs and spiritual aspirations of a patient with a psychiatric disorder require heightened awareness on the part of mental health professionals.

### 1.4 Aim and hypotheses

The brief literature review in 1.1-1.3 shows the disintegrating effect of schizophrenia on the personal value system. However, the contribution of patients’ individual religious faith to this process remains to be identified.

The bulk of modern-day research on the role of religion and spirituality in schizophrenia focuses mostly on *correlations* between religious faith (as a complex characteristic attributed to the patient) and the nature of the mental illness, its symptoms, and the outcomes of therapy and rehabilitation practices. Studies also examine positive and negative religious coping as a response mechanism to stressful life events. However, insufficient attention has been paid to the structure and composition of PVSs among believers with severe mental disorders.

The lack of credible data in this area prompted us to conduct an empirical study, *aimed* at identifying the contribution of religious faith to the personal value system (the structure and composition of its elements) in patients with schizophrenia.

Our general *hypothesis* was that the religious faith of patients with schizophrenia contributes to the preservation of their personal value system. Accordingly, we hypothesized the following indicators of such preservation:

1. personal values would be more interrelated in religious patients than in non-religious patients;
2. the structure and combination of personal values in religious patients and healthy believers would be similar;
3. the structure and combination of personal values in religious patients would differ from the structure and combination of values in nonreligious patients and healthy nonbelievers.

## 2 Method

### 2.1 Research design

For the study, a factorial design (2x2) was used, including two conditionally independent variables: 1 – mental illness (absent or present); 2 – religious faith (absent or present). We compared personal value systems of participants (N = 65) divided into four groups: mentally ill believers of Orthodox Christian faith, mentally ill non-believers, healthy believers of Orthodox Christian faith, and healthy non-believers.

### 2.2 Participants

The study involved four groups of participants:

- Religious patients (RP): 24 individuals (9 males, 14 females, average age = 26.7 ± 6). The group included patients of Orthodox faith suffering from paroxysmal forms of schizophrenia and discharged from Mental Health Research Center in Moscow, Russia, after they underwent treatment for an acute psychotic episode combined with mental automatism, paranoid delusions and hallucinations, or a major schizoaffective or delusional disorder. At the time of the study, the participants had been in persistent drug-controlled remission for a minimum of 1 year and were perfectly capable of fulfilling the requirements imposed by the method. The key criterion to assign respondents to this group was the fact that they had faith in God long before the onset of schizophrenia.
- Non-religious patients (NP): 12 individuals (3 males, 9 females, average age = 28.9 ± 7.6). The sample included patients diagnosed with schizophrenia who did not adhere to any religion and exhibited clinical symptoms similar to those observed in the group of Orthodox patients.
- Healthy believers (HB): 15 individuals (4 males, 11 females, average age = 30.2 ± 6.4). The sample included practicing believers of Orthodox faith with no mental health issues.
- Healthy non-believers (HN): 14 individuals (5 males, 9 females, average age = 29.7 ± 5.1). The group consisted of healthy participants professing no faith in God or any other spiritual attitudes.

The study is approved by Psychological and Pedagogical Research Ethics Committee at Moscow City University, Russia (Number 008-2018, 15.03.2018). All subjects gave informed consent to participate in the study.

### 2.3 Stimulus material, tools, measures, and procedure

To study personal value systems, we applied a methodological approach, tools, measures, and procedures based on Kelly’s *theory of personal constructs* (29). According to Kelly, personal constructs are unique cognitive structures formed by two opposite poles that reflect the subjective significance of phenomena for an individual. Each person has a different set of personal constructs. In operational terms, personal constructs function as bipolar scales of subjective evaluation (‘good – bad’, ‘interested – not interested’, etc.) of various objects – *‘elements’*, according to Kelly’s terminology (29). This constructivist approach can be applied to analyzing one’s personal value system, as it provides researchers with the opportunity to reach to the deepest, tacit systems of meaning a person is barely aware of (13).

Kelly’s methodology has a good track record in studies of patients with schizophrenia (see, e.g., 8, 19). The implementation of this methodology in the present study involved the following four sequential steps.

First, a *stimulus material* universal for all subjects was developed. This material was a set of items (‘elements’) for subsequent quantitative subjective evaluations. The set of items included a list of 16 value statements which operationalized generalized and potentially significant values for the participants.

Second, personal constructs in each subject were identified as individual scales of subjective evaluation. To elicit surface-level constructs (those that are easy to realize), we employed Kelly’s *triad method (the Minimum Context Card Form)*. To elicit deep-level constructs (those that are poor to realize), we utilized *Hinkle’s laddering method*.

Third, we identified the subjective evaluations of each participant with respect to the presented ‘elements’ (value statements) within their sets of personal constructs. This task was accomplished using the *repertory grid method* (Kelly’s *grid using ratings*).

Fourth, the structure and combination of personality values within each of the four participant groups was investigated through correlation and factor analyses.

The subjective evaluations of value statements provided by the participants were a measure of the subjective significance of specific values within their PVSs. The structure of the PVSs was operationalized through a set of correlations between values (value statements). The factors identified through factor analysis were considered to be combinations of values (meta-values) or most generalized meanings in PVSs.

The *stimulus material* was represented by 16 value statements reflecting generalized values (see **Table 1**). These statements reflect a wide range of individuals’ values concerning different aspects of their lives and are relevant to the subjects and objectives of this study. Among others, these value statements address health and religion. Initially, after reviewing various classifications of values and available methods of measuring personal values, we compiled a comprehensive list of 151 value statements. This list was then summarized and reduced to 32, and then after pre-testing on a sample of 40 people, to 16 value statements.

**Table 1:** Value statements – ‘elements’ used to identify the composition of personal value systems.

No.	Value statements	Generalized values
1	Do no harm to any living thing	Higher moral values
2	Strive for spiritual development	Spirituality
3	Find the purpose and meaning of your existence	Meaningful life
4	Create new things, live creatively	Creativity
5	Be healthy (restore, preserve, enhance, obtain health)	Health
6	Be confident in your future (see the world as a safe place)	Confidence in one’s future
7	Communicate with friends and family	Affiliation
8	Treat yourself well (have positive self-esteem)	Self-respect
9	Maximize your skills and abilities	Self-realization
10	Achieve family well-being, build harmonious relations with friends and family members	Family well-being
11	Improve your living conditions	Better housing conditions
12	Achieve professional success and desired social status	Professional and social success
13	Achieve material well-being	Material well-being
14	Be able to get along with people	Social interactions, care for others
15	Be sensitive to the needs of other people	Social interactions, care for others
16	Seek to be with God	Religious aspirations

The Triad Method: The Minimum Context Card Form (15, 16). In order to identify surface-level personal constructs by the triad method, each participant was presented with 3 cards featuring 3 value statements (‘elements’) out of 16, repeatedly and in different order, until all the 16 statements appeared at least once. The respondent was asked to name a significant attribute or quality that made two of the three value statements *similar* to each other (one pole of the construct) and *different* from the third one (the other pole of the construct). Then the experimenter would record the two poles of the studied construct.

Hinkle’s Laddering Method (15, 16). This tool helped explicate tacit, subconscious deep-level personal constructs – those that a person can realize through a special testing procedure. The participant was asked a question regarding the two poles of each surface construct that had been previously identified: “What is more important to you:… (one pole of the construct) or… (the other pole of the construct)?”. After receiving a response, each participant was asked to explain reasons or goals attached to a surface-level construct, with questions like “Why is it so important for you to…?”, “Why do you think is it necessary to…?” Once answers were given and recorded as a second-order construct (with only one pole so far), we repeated the questions in relation to these answers to get to a third-order construct. Our objective was to identify the ultimate, deepest construct – the final point in the participant’s chain of responses. The procedure continued until the respondent could not or did not want to generate new constructs, or until the respondent repeated a previously named, more surface construct as a response. The last named deep construct was regarded as the *ultimate construct.* Then the same technique was applied to other surface-level constructs. Subsequently, we asked the participant to report what was the opposite (pole) for each of the ultimate constructs that had been previously identified. The answer was recorded.

The Repertory Grid Method: A Grid Using Ratings (15, 16). The ultimate constructs identified in the subject served as subjective rating scales with two opposite poles. According to instructions, the participant was supposed to use an 11-point scale (one pole of the construct is 1, the opposite pole of the construct is 11) to give ratings to each of the 16 ‘elements’ (value statements). Given that each participant possessed a *set* of constructs, each of the 16 value statements was evaluated as many times as the number of constructs identified in the participant. As personal constructs are unique and largely unpredictable, in order to be able to compare the data coming from different respondents, we introduced *three additional constructs with contrasting poles* and offered them as additional scales to all the participants (see **Table 2**). This approach enabled us to obtain three additional ratings of each value statement from each participant. If a value statement was deemed by a participant to be irrelevant to a specific construct and could not be adequately evaluated, it received a score of ‘6’, thereby occupying a position in the middle of two poles of the scale. The participant’s scores were entered into a table (‘grid’), the columns of which corresponded to the 16 value statements, and the rows corresponded to the scales (constructs).

**Table 2:** Additional bipolar personal constructs provided to the participants.

№	Pole of the construct	Pole of the construct
1	Meaningfulness, purposefulness	Meaninglessness, randomness
2	Possible harm to others	No harm to others
3	Does matter to me	Does not matter to me

The data obtained by the repertory grid method were subjected to statistical processing. Correlation analysis using the Pearson correlation coefficient was applied to each group of respondents in order to identify interrelations between the value statements. Subsequently, exploratory factor analysis was applied. Relevant factors were selected according to the Kaiser criterion, and the selected factors were subjected to Varimax rotation. The statistical analysis employed in this study, particularly factor analysis, necessitates a large number of observations within the sample. Typically, the sample size is equivalent to the number of subjects in the study. However, in the present study, the sample size corresponded to the aggregate of *all ultimate constructs* identified in the participants within one group. This was due to the fact that each participant repeatedly rated the 16 value statements using not one but a set of subjective scales (constructs).

The descriptions of the tools and the sequence of their application that have been given generally provide information about the research procedure. In addition, it should be noted that the work with each participant was conducted in the absence of time constraints, with necessary breaks. The total time of examination for one subject averaged 3.5–4 hours.

## 3 Results

### 3.1 Correlation analysis

**Figure 1:**
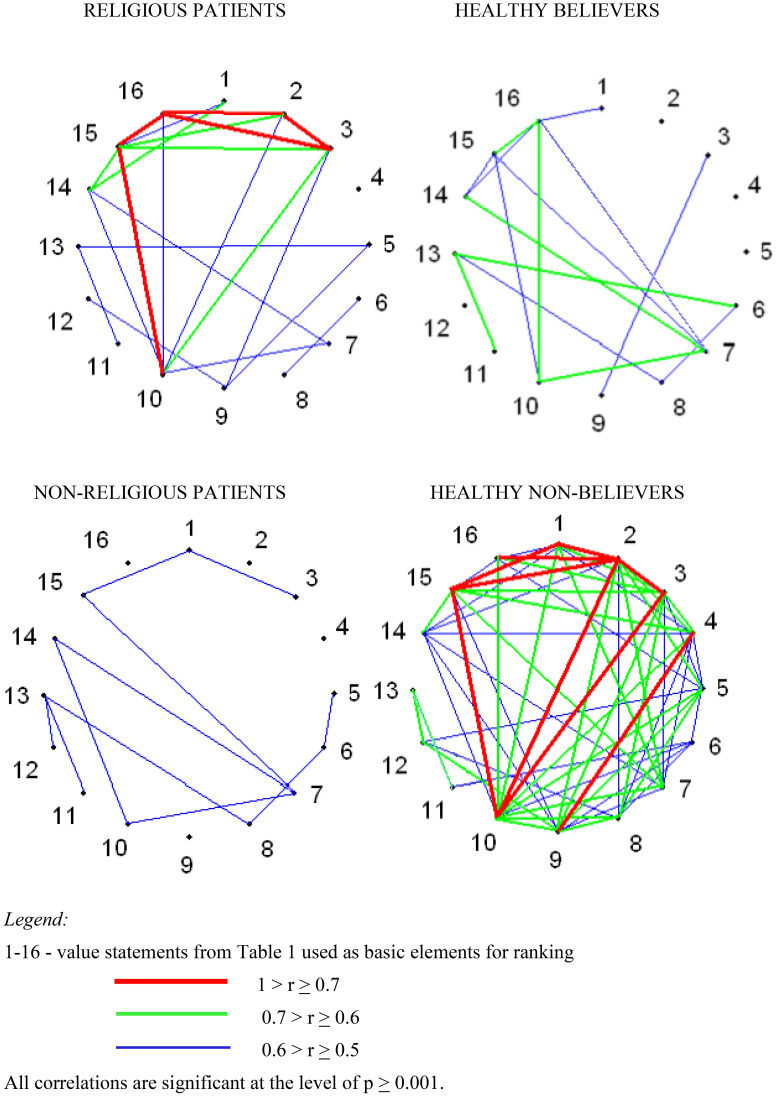
Correlation network of personal value systems in different groups of respondents.

The results of the correlation analysis are displayed as correlation matrixes for each of the four groups of participants (**Tables 3**–**6** in **Supplementary Material**). The data are also visualized in **Figure 1**, which demonstrates significant differences between the groups. The figure illustrates correlations between the 16 value statements that the respondents rated using personal constructs.

We should specify that our study does not look for correlations between *personal constructs*; it focuses on correlations between *value statements* rated using the constructs, which is also a common practice in mental health research. In this case, correlation analysis provides information on the extent of differentiation and integration of participants’ PVSs.

To comprehend the psychological significance of the indicator ‘number of correlations’, it is necessary to refer to the field of personal constructs research. Researchers regard the number of correlations between constructs or the number of independent constructs in an individual as indicators of ‘cognitive differentiation’. The concept of ‘cognitive differentiation’ is interpreted as a measure of the complexity and multidimensionality of a person’s perception of a certain area of experience (15). A large number of correlations indicates the undifferentiated nature of this perception – constructs overlap and are combined into a minimal number of factors. Conversely, a minimal number of correlations points to a lack of integration within perception. In the context of our study, the number of correlations reflects the measure of ‘value differentiation’. An extensive number of correlations suggest a lack of differentiation of PVS, and vice versa. However, when analyzing ‘value differentiation’, it is crucial to consider the overall structure of the correlations. This structure indicates the extent to which values are integrated (correlations are combined into factors) or fragmented (correlations are scattered).

Personal value systems in religious patients with schizophrenia and in healthy respondents who are Orthodox believers have a lot in common. However, despite the similarity in the structures of values observed in both groups, the PVSs of religious patients demonstrate a slightly higher degree of integration and enhanced ‘concentration’ of values compared to those of healthy believers. By ‘concentration’ we mean an increase in the strength of correlations. From a psychological perspective, this could indicate that values that are closely related share a common source of meaning.

There are significant discrepancies in interrelationships among values of healthy non-believers and non-religious patients with schizophrenia. The values of healthy non-believers are massively interrelated, much more than in all other groups of subjects. This value structure indicates a certain degree of integration in the PVS, while also suggesting a low differentiation. However, the number and strength of correlations in the group of non-religious patients is much lower than in the other samples. The value structure exhibited by these patients suggests a fragmentation of their PVSs, rather than its high differentiation.

### 3.2 Factor analysis

**Figure 2:**
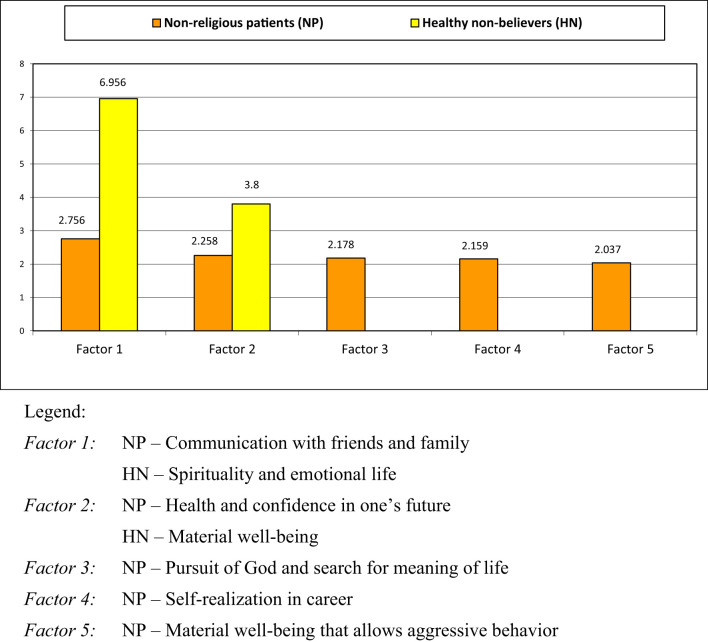
Factors representing meta-values (superordinate values) in non-religious patients and healthy believers.

**Figure 3:**
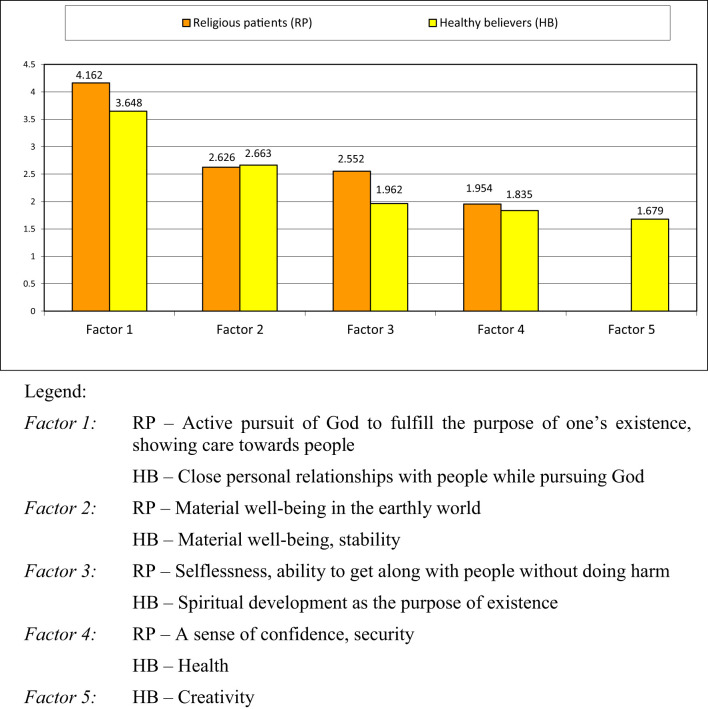
Factors representing meta-values (superordinate values) in religious patients and healthy believers.

The results of the factor analysis are presented in **Figures 2**, **3**.

#### 3.2.1 Non-religious patients with schizophrenia (NP) and healthy non-believers (HN)

The first thing that comes out of analyzing factors in the four groups is a strikingly small number of factors identified in healthy non-believers (the HN group) in comparison with other groups. The first factor – ‘Spirituality and emotional life’ as a meta-value (superordinate value) – is manifested in many value statements, including: [2] *spiritual aspirations*, [15] *social interactions, care for others (sensitivity to the needs of other people)*, [1] *higher moral values*, [3] *meaningful life*, [10] *family well-being*, [16] *religious aspirations*, [4] *creativity*. All these elements have a factor loading higher than 0.7, with spirituality and religious aspirations being secular concepts, as understood by non-religious respondents. The value statement [5] *health*, along with some others, also falls into this factor, though it apparently strays from being a purely physical notion into something more spiritual or emotional. The second factor – ‘Material well-being’ – in the HN sample predictably combines such value statements as [13] *material well-being*, [11] *improvement of housing conditions* and [12] *professional and social success*, with factor loading higher than 0.7.

The five factors identified for the NP sample indicate that the composition and structure of PVSs in non-religious patients with schizophrenia differ qualitatively and quantitatively from PVSs of non-believing healthy participants. The main peculiarity of the arrangement of these factors and corresponding meta-values is that they are all equal in weight, with no *leading factor*. The first factor for this group is ‘Communication with relatives and friends’, the second factor is ‘Health and confidence in one’s future’, and Factor 3 is ‘Pursuit of God and search for the meaning of life’. Other factors are marked a ‘Self-realization in career’ and ‘Material well-being that allows aggressive behavior’.

#### 3.2.2 Religious patients with schizophrenia (RP) and healthy believers (HB)

The two groups of religious participants have a similar set of meta-values (superordinate values): the RP group scored 4 factors, while the HB group recorded 5 factors. The leading factors (Factor 1) for both samples relate to faith in God and are significantly weightier than the other ones. At the same time, Factor 1 of the RP group stands taller above the other factors if compared with Factor 1 of the HB group. Factor 2 corresponds with the meta-value ‘Material well-being’ and has almost equal weight in both groups. Factor 4 also has similar weights in the RP and the HB samples, signifying ‘A sense of confidence and security’ and ‘Health’ respectively, while Factor 5 ‘Creativity’ of the HB group is small in weight. When comparing the groups of religious patients and healthy religious individuals, it is easy to notice that their biggest factors (Factor 1 and Factor 2) are very alike – in terms of both value-meaning constructs and their place in the factor hierarchy – while their second most important factors (Factor 2) also have similar weights.

The groups of religious respondents start diverging beginning with Factor 3. For the RP sample Factor 3 stands for the meta-value of ‘Selflessness and ability to get along with people without doing harm’, while for the HB group Factor 3 reads as ‘Spiritual development as the purpose of existence’.

## 4 Discussion

The results of the correlation analysis demonstrate that the presence/absence of a mental disorder, such as schizophrenia, makes a differential contribution to the personal value systems of religious and non-religious individuals. Mental disorder contributes to the number and strength of connections between individual values in Orthodox believers, however, it does not significantly contribute to the transformation or disintegration of their personal value systems. It is likely that enhanced integration and mutual rapprochement (‘concentration’) of individual values in religious patients with schizophrenia is a prerequisite for coping with the adverse effects of the mental disorder. In contrast, the PVSs of non-religious patients are indicative of low integration of values and fragmentation. This finding aligns with the conclusions of previous studies (36, 60). The seeming ‘value differentiation’ observed in non-religious patients may be explained, in analogy with ‘cognitive differentiation’, by the inconsistency of repertory grid scores that has long been identified in patients with schizophrenia (see 15, 17). Consequently, religious patients with schizophrenia exhibit *more intact* PVSs compared to their non-religious counterparts.

The contribution of religious faith to personal value systems varies significantly between healthy individuals and patients with schizophrenia. Healthy non-believers exhibit a greater number of connections between values and a different combination of values compared to healthy Orthodox believers. At the same time, the opposite picture emerges when analyzing the differences in the PVSs between mentally ill patients – religious and non-religious.

Correlation analysis leaves open the following questions. Should the higher relatedness of personal values in healthy non-believers be regarded as evidence of *optimal* integrity and differentiation of the PVS? Furthermore, should fewer interrelationships between values in healthy believers be interpreted as a sign of structural complexity of their PVSs or violation of its integrity? According to Norris et al., a healthy person would typically exhibit multiple well-differentiated clusters of personal constructs that are independently connected to one another (42). In contrast, the personal constructs of patients with obsessive neurosis are either excessively monolithic (meshed into one large cluster) or fragmented (forming many small, unrelated clusters) (42). To address the inquiries raised, we will turn to the findings of the factor analysis.

A small number of factors in participants from the HN sample be indicative of poorly differentiated, ‘black-and-white’ personal value systems, with individuals relying on a *single* meta-value to assess significance of *various* aspects of life. Within this group, we identified two meta-values that are diametrically opposed to each other: ‘Spirituality and emotional life’ and ‘Material well-being’. This finding suggests that the behavior of healthy non-believers, when governed by values, is ultimately aimed at obtaining material or spiritual (emotional) benefits. Consequently, well-integrated PVSs may not be as optimal as they might seem, since poor differentiation may signify limited sources of meaning.

The absence of a leading factor – the ‘core’ of the personal value systems – in non-religious patients with schizophrenia, in conjunction with the fragmented interrelationships between individual values, as shown in **Figures 1**, **2**, may be evidence of them being largely unaware or confused about their values, dispositions and attitudes, as well as evidence that PVSs in these individuals are inconsistent and poorly organized. These findings align with the results of several studies that have shown that PVSs are narrow and distorted in patients with schizophrenia or other severe forms of psychiatric pathology (60, 63).

This pattern appears to be attributable to the influence of mental disorder. The most prominent characteristic of the NP group is the longing of affected individuals to get support from their family and friends. In their state, it is natural to exhibit increased (in comparison with healthy individuals) concern for one’s health and to connect [5] *health* to [6] *confidence in one’s future*. The values identified in non-religious patients, as outlined in this study, align with those reported in another study by Leontieva (35): *family, health, material security*, etc. This finding suggests that we are dealing with an objective tendency.

The presence of Factor 3 ‘Pursuit of God and search for the meaning of life’ merits separate discussion. Mental illness can profoundly disrupt an individual’s ability to continue living as they once did (8, 26, 36). The onset of schizophrenia in particular can lead to the dissolution of previously held life goals and a reevaluation of purpose (‘crisis of meaning in life’). This can result in individuals seeking new meaning in life and purpose in the face of these challenges. In this quest for purpose and meaning in life, patients with schizophrenia often turn to the Divine, despite not identifying as religious. However, the religious aspirations of the NP respondents appear to be abstract in nature, as they do not entail a pursuit of paths toward God offered by religion; the individuals do not seek to obtain any real experience of God or communion with God. Factor 4 ‘Self-realization in career’ is likely a means of maintaining connection with society, which is a relevant and challenging task for mentally ill individuals. Finally, the meta-value ‘Material well-being’ is expressed in the NP group by Factor 5, which also allows aggressive behavior as a means of achieving it.

In the case of religious patients and healthy believers, the presence of leading factors with significant weights as well as the overall interconnectedness of values presented in **Figure 1** can be interpreted as evidence of the complexity of personal value systems in these respondents. Given the observed similarity in structure and content of these systems, a cautious conclusion can be drawn that *schizophrenia may not have a destructive effect on the personal value system in individuals with a religious worldview as it allows the preservation of its key elements.*

Nonetheless, it could be assumed that a specific transformation of the PVSs in religious individuals does take place. We observe the groups of HB and RP diverging on Factor 3: the meta-value of ‘Spiritual development as the purpose of existence’ aligns with RP, while ‘Selflessness and ability to get along with people without doing harm’ corresponds to HB. Hypothetically, one could speak of a shift from the basic value of spiritual growth to the value of kind, altruistic and respectful social relationships. This shift likely reflects a general tendency to place greater importance on the social environment and social support in patients with schizophrenia (35, 44).

It is noteworthy that Factor 3 of the NP group (non-religious patients) aligns with certain factors in the RP and HB groups, specifically ‘The pursuit of God and the search for the meaning of life’. However, the personal values underlying these factors exhibit significant divergence. For the religious participants, values of religious aspirations and spiritual development are not abstract concepts; rather, they stem from their personal experience of communion with God interpreted within the context of the Orthodox doctrine. The meta-value ‘Selflessness and ability to get along with people without doing harm’ underscores the social orientation of the value elements of Orthodox patients with schizophrenia emphasizing the significance of *peaceful* social interactions for the RP respondents (cf. the acceptance of *aggressive* behavior as Factor 5 of the NP group).

It is noteworthy that the meta-value ‘Health’ does not emerge as a separate factor in the RP group (religious patients). However, the value [5] *heath* is included in Factor 2 ‘Material well-being in the earthly world’ with the weight of 0.659. At the same time, ‘Health’ serves as a fundamental value (a separate factor) for non-religious patients with schizophrenia.

We suggest that these differences can be attributed to the divergent health attitudes exhibited by these groups of respondents. For non-religious patients [5], *health* is closely aligned with the values of [6] *confidence in one’s future* and [8] *self-respect*. These respondents conceptualize health as a key condition that determines their future life in general and their self-acceptance. Conversely, the attitudes of religious patients towards health are characterized by a distinct perspective, largely influenced by the Christian Orthodox conception of illness and health. In the context of Orthodox doctrine, the fundamental importance of the spiritual aspect of illness is asserted. According to this perspective, illness is not perceived as an evil that hinders a full life, but rather as an opportunity for spiritual growth and the adoption of a righteous lifestyle. Orthodox Holy Fathers’ writings affirm that illness is not necessarily a consequence of sin (34). Sometimes, illness is regarded as an outcome of God’s divine providence, which guides individuals toward positive moral transformation and salvation. Orthodox theologians emphasize not the causes of illness, but rather what the Orthodox believer’s perception of it should be, its *meaning*. Therefore, illness and healing must be accompanied by a spiritual rethinking of one’s life by the believer (2). It is important to note that healing encompasses more than merely the restoration of bodily functions. In the context of the New Testament, as Lavrentiev observes (34), healing is regarded as salvation or the beginning of salvation, representing the re-establishment of a fractured connection with God. Consequently, for religious patients, illness is not perceived as an impediment to achieving life goals or as a determiner of life’s meaning. As Schnell notes, the ability to comprehend the meaning of illness often facilitates more effective coping mechanisms, but this is a long-term process involving regaining control over one’s life (52).

Analyzing general trends, we can outline the following picture.

Schizophrenia as a factor contributes significantly to the disintegration of an individual’s personal value system. The system of values of a healthy non-religious person, the core of which consists of meta-values – spiritual and material – in the conditions of the disorder has a different form. In this case, the values that are typically important for a healthy person are rendered less significant, the links between them are disrupted, and the key values are those associated with the disease: ‘Communication with friends and family’, ‘Health and confidence in one’s future’.

A religious person, specifically an Orthodox believer, holds a different system of values, which is presumably more differentiated than that of a non-believer, and combines meta-values: ‘Close personal relationships with people while striving for God’, ‘Material well-being, stability’, ‘Spiritual development as the purpose of existence’, ‘Health, and ‘Creativity’. In the context of schizophrenia, the personal value system of the believer remains broadly the same, despite slight differences. This could be explained by the attitude toward illness as a form of trial, which is integrated within the framework of his or her religious worldview. In this way, as previously mentioned, illness is not regarded as a hindrance to achieving life goals and personal meanings inspired by religion.

The patterns identified in the present study can be discussed in the context of the psychology of meaning in life because personal value systems are nourished from specific sources of meaning. Schnell empirically elicited 26 major sources of meaning in life, which were grouped into four categories: ‘Selftranscendence’, ‘Selfactualization’, ‘Order’, and ‘Well-being and relatedness’ (52).

The non-religious patients in our study appear to be facing a crisis of meaning. This is follows from their fragmented PVS, the absence of a value ‘core’, and their pursuit of Divine and search for the meaning of life. However, there is insufficient evidence to conclude that they are devoid of meaningfulness. First, the crisis of meaning and meaningfulness are not completely opposite (52). Second, these individuals prioritize the value groups (meta-values) such as ‘Communication with relatives and friends’, ‘Health and confidence in one’s future’, ‘Self-realization in career’, etc. While the extent to which these values are actualized in behavior remains unclear, they do point to potential sources of meaning for non-religious patients. These sources include *community*, *care* (‘Well-being and relatedness’), *health*, sp*irituality* (‘Selftranscendence’), *achievement* (‘Selfactualization’) (see 52).

Healthy non-believers, whose two-factor PVS consists of the meta-values ‘Spirituality and emotional life’ and ‘Material well-being’, appear to be characterized by a broader set of sources of meaning. This can be inferred from the diversity of values combined in each factor. In addition, the healthy non-religious group is the most heterogeneous group in the study, in contrast to the other groups, where participants are united either by psychiatric diagnosis, common Orthodox faith, or both. Consequently, a group of healthy non-believers should be characterized by significant individual differences in meaningfulness and sources of meaning. This heterogeneity within the atheist sample, and between atheists and ‘nons’ (‘not church members’), was noted by Schnell and Keenan (53).

Similar PVSs in religious patients and healthy believers of the Orthodox faith demonstrate that the key sources of meaning for them are *explicit religiosity*, *spirituality* (‘Vertical Selftranscendence’), and sources from the ‘Well-being and relatedness’ category. It should be recalled that in Orthodox patients the value of *health* did not emerge as a discrete factor when compared to non-religious patients and healthy believers. Instead, it was loaded on the factor ‘Material well-being in the earthly world’, suggesting that this value possesses a distinct and non-global significance for them. In light of Park’s (46) model of global and situational meanings, it can be posited that in cases of schizophrenia, religion contributes to the maintenance of global meanings and, consequently, the preservation of PVS.

The findings of the study suggest that both our general and particular hypotheses are confirmed in a Russian sample. Indeed, the religious faith of Orthodox patients with schizophrenia has been demonstrated to contribute to the preservation of their personal value system. The present study’s findings align with those of previous research, which identified a positive correlation between religiosity and the quality of life and psychosocial functioning of patients diagnosed with schizophrenia (5, 9, 12, 27, 39, 41).

However, the study we have conducted certainly has a number of *limitations*. It is important to note that the validity of our findings is restricted to patients in whom religiosity is not an inherent component of the patient’s mental disorder, but rather, is utilized as a psychological resource. Moreover, the findings are limited by different contexts.

First, there is the *religious context*. The believing participants of the study were adherents of Orthodox Christianity. This must be taken into account when interpreting the results, as Orthodox Christianity is a traditional religion with a specific value view of human being, life goals, ways to achieve them, and the nature of suffering in the world. The Orthodox religion possesses a comprehensive array of tools (e.g., religious injunctions, explanations, examples of behavior) for conceptualizing and making meaning of illness, suffering, and living in these conditions. This view affects the formation of adherents’ personal value systems. Therefore, the patterns identified in this study may not be replicable in adherents of other traditional religions and new religious movements, as well as in individuals who exhibit modern forms of religiosity (e.g., ‘believing without belonging’ (11), ‘free-floating believers’ (22), ‘patchwork religion’ (61), etc.). A similar consideration applies to non-religious participants of the study, who may demonstrate significant variations in their personal value systems and attitudes toward religion (ranging from indifference to militant atheism).

Second, it is imperative to consider the *cultural context*. The present study exclusively involved representatives of Russian culture, thereby implying a specific value background inherent to it. To illustrate this point, we can turn to Schwartz’s theory of values. According to this theory, if values such as Autonomy, Egalitarianism, and Harmony are of particular importance to representatives of Western Europe, then for Russians, values such as Embeddedness, Hierarchy, and Mastery assume greater significance (55). These distinctions may prove to be significant in the subsequent verification of the patterns that have been identified.

Another important limitation of the study is the cross-sectional design, which does not provide a foundation for causal inferences. Furthermore, the study was underpowered because of the limited number of participants. Increasing the number of participants would provide a more robust evidence base. The preponderance of female subjects in all groups also requires closer examination. It should be noted that gender differences were not considered in this study. However, it is acknowledged that women’s religiosity differs from men’s. Thus, a more nuanced, gender-sensitive approach is required to elucidate the role of religious faith in preserving the personal value system of patients with schizophrenia.

We recognize that the study does not provide sufficient evidence for firm clinical recommendations. Nevertheless, the findings may hold some relevance for clinical practice. A promising direction in the rehabilitation of religiously affiliated patients diagnosed with schizophrenia is the currently developing confessional-oriented approach (20, 33). The primary objective of this approach is to promote the development of positive religious coping mechanisms. When implementing this approach, in our opinion, it is essential to consider the patients’ personal value systems, particularly their spiritual values, and sources of meaning in life. In accordance with the classification of religious coping strategies (44), the following strategies in rehabilitation could presumably be applicable when implementing the confessional-oriented approach in working with Orthodox patients with schizophrenia:

1. Conserving the fundamental meaning of life, based on the Orthodox Christian values of the patient.
2. Providing social support to the patient through the Orthodox community. The foundation for this line of work is rooted in the meta-value of Orthodox patients with schizophrenia – ‘Selflessness and ability to get along with people without doing harm’. This fundamental value, reflecting the key Christian principle of ‘Love your neighbor as yourself’ (Matt. 22:39), underscores the social orientation of patients and the significance of peaceful relations for them.
3. Transformational coping – rethinking life situations and one’s own identity in the context of Orthodox Christianity. This strategy involves reevaluating negative events from a benign perspective, whereby these events are given a potentially beneficial significance as having been allowed by God to correct, improve, and guard against mistakes and falls. In this context, it is imperative to facilitate the actualization of the concepts of ‘strengthening the spirit through the weakness of the flesh’ and ‘unconditional acceptance and care from God’ rather than the concept of ‘punishment for sins’.
4. A strategy for gaining the emotional comfort that religious faith provides (comfort, solace, forgiveness, reconciliation with oneself).

The strategies described should be considered as assumptions concerning the possibilities for clinical work. Determining the conditions, limits of applicability and effectiveness of these strategies is a task for future research.

Finally, given all the limitations, the present research should be regarded as a pilot study whose results need further validation and refinement and whose findings require additional evidence. To that end, a more extensive and robust study across a range of religious and cultural contexts is necessary. In order to trace causal relationships in the future, it would be desirable to employ a longitudinal rather than a cross-sectional design. Subsequent studies should also aim to identify the personal value systems of those patients with schizophrenia who primarily use negative religious coping mechanisms.

## Data Availability

The datasets presented in this article are not readily available due to legal restrictions on the use of personal data from patients at the treatment facility. Requests to access the datasets should be directed to the corresponding author.
