# Supplementary material for: Does religious faith contribute to the preservation of personal value system in patients with schizophrenia? an empirical research
Source: Front Psychiatry. 2025 Apr 7;16:1553990. doi: 10.3389/fpsyt.2025.1553990 (PMC12009826; doi:10.3389/fpsyt.2025.1553990)
Supplement: Supplementary file 1 [file Table1.docx]

Supplementary Material

# Supplementary Tables

*Table 3. RELIGIOUS PATIENTS: the matrix of inter-correlations of value statements*

| **Value**  **statements** | **1** | **2** | **3** | **4** | **5** | **6** | **7** | **8** | **9** | **10** | **11** | **12** | **13** | **14** | **15** | **16** |
| --- | --- | --- | --- | --- | --- | --- | --- | --- | --- | --- | --- | --- | --- | --- | --- | --- |
| **1** | 1.00 |  |  |  |  |  |  |  |  |  |  |  |  |  |  |  |
| **2** | 0.41 | 1.00 |  |  |  |  |  |  |  |  |  |  |  |  |  |  |
| **3** | 0.32 | 0.71* | 1.00 |  |  |  |  |  |  |  |  |  |  |  |  |  |
| **4** | 0.27 | 0.43 | 0.41 | 1.00 |  |  |  |  |  |  |  |  |  |  |  |  |
| **5** | 0.39 | 0.30 | 0.30 | 0.27 | 1.00 |  |  |  |  |  |  |  |  |  |  |  |
| **6** | 0.07 | 0.19 | 0.18 | 0.36 | 0.37 | 1.00 |  |  |  |  |  |  |  |  |  |  |
| **7** | 0.40 | 0.22 | 0.18 | 0.36 | 0.46 | 0.45 | 1.00 |  |  |  |  |  |  |  |  |  |
| **8** | -0.04 | -0.07 | -0.12 | 0.28 | 0.23 | 0.53* | 0.24 | 1.00 |  |  |  |  |  |  |  |  |
| **9** | 0.39 | 0.48 | 0.53* | 0.48 | 0.55* | 0.22 | 0.35 | 0.19 | 1.00 |  |  |  |  |  |  |  |
| **10** | 0.44 | 0.57* | 0.63* | 0.46 | 0.39 | 0.36 | 0.51* | 0.00 | 0.45 | 1.00 |  |  |  |  |  |  |
| **11** | 0.11 | -0.03 | -0.01 | 0.26 | 0.41 | 0.25 | 0.28 | 0.22 | 0.22 | 0.06 | 1.00 |  |  |  |  |  |
| **12** | 0.18 | 0.07 | 0.20 | 0.26 | 0.40 | 0.19 | 0.30 | 0.36 | 0.53* | 0.25 | 0.21 | 1.00 |  |  |  |  |
| **13** | 0.14 | -0.05 | -0.06 | 0.16 | 0.51* | 0.33 | 0.32 | 0.38 | 0.25 | 0.15 | 0.58* | 0.43 | 1.00 |  |  |  |
| **14** | 0.61* | 0.42 | 0.37 | 0.26 | 0.42 | 0.16 | 0.50* | -0.13 | 0.29 | 0.58* | 0.19 | 0.19 | 0.19 | 1.00 |  |  |
| **15** | 0.58* | 0.67* | 0.63* | 0.38 | 0.26 | 0.10 | 0.39 | -0.17 | 0.44 | 0.71* | -0.10 | 0.14 | -0.03 | 0.66* | 1.00 |  |
| **16** | 0.49 | 0.82* | 0.70* | 0.37 | 0.30 | 0.10 | 0.21 | -0.13 | 0.48 | 0.58* | -0.07 | 0.11 | -0.16 | 0.45 | 0.72* | 1.00 |

*Note:* * p < 0.001

*Table 4. HEALTHY BELIEVERS: the matrix of inter-correlations of value statements*

| **Value**  **statements** | **1** | **2** | **3** | **4** | **5** | **6** | **7** | **8** | **9** | **10** | **11** | **12** | **13** | **14** | **15** | **16** |
| --- | --- | --- | --- | --- | --- | --- | --- | --- | --- | --- | --- | --- | --- | --- | --- | --- |
| **1** | 1.00 |  |  |  |  |  |  |  |  |  |  |  |  |  |  |  |
| **2** | -0.06 | 1.00 |  |  |  |  |  |  |  |  |  |  |  |  |  |  |
| **3** | 0.04 | 0.46 | 1.00 |  |  |  |  |  |  |  |  |  |  |  |  |  |
| **4** | -0.10 | 0.43 | 0.19 | 1.00 |  |  |  |  |  |  |  |  |  |  |  |  |
| **5** | 0.05 | 0.15 | 0.23 | 0.09 | 1.00 |  |  |  |  |  |  |  |  |  |  |  |
| **6** | -0.12 | -0.03 | 0.02 | -0.08 | 0.26 | 1.00 |  |  |  |  |  |  |  |  |  |  |
| **7** | 0.36 | 0.19 | 0.06 | 0.07 | 0.22 | -0.03 | 1.00 |  |  |  |  |  |  |  |  |  |
| **8** | -0.10 | 0.12 | 0.05 | 0.17 | 0.05 | 0.52* | 0.08 | 1.00 |  |  |  |  |  |  |  |  |
| **9** | -0.10 | 0.28 | 0.50* | 0.44 | 0.40 | -0.14 | 0.08 | 0.09 | 1.00 |  |  |  |  |  |  |  |
| **10** | 0.37 | 0.04 | 0.21 | -0.11 | 0.39 | 0.06 | 0.66* | -0.18 | 0.07 | 1.00 |  |  |  |  |  |  |
| **11** | -0.24 | -0.01 | -0.03 | 0.24 | 0.23 | 0.36 | 0.03 | 0.31 | 0.27 | -0.17 | 1.00 |  |  |  |  |  |
| **12** | -0.08 | 0.26 | 0.42 | 0.19 | 0.37 | 0.23 | 0.09 | 0.46 | 0.47 | 0.19 | 0.26 | 1.00 |  |  |  |  |
| **13** | -0.23 | -0.05 | -0.02 | 0.02 | 0.44 | 0.64* | 0.16 | 0.54* | 0.06 | 0.09 | 0.60* | 0.43 | 1.00 |  |  |  |
| **14** | 0.42 | 0.21 | -0.01 | 0.18 | 0.03 | -0.40 | 0.66* | -0.07 | 0.10 | 0.48 | 0.02 | 0.01 | -0.17 | 1.00 |  |  |
| **15** | 0.43 | 0.21 | 0.06 | 0.00 | -0.03 | -0.07 | 0.53* | -0.12 | -0.13 | 0.50* | -0.03 | -0.09 | -0.06 | 0.55* | 1.00 |  |
| **16** | 0.52* | 0.12 | 0.09 | -0.05 | -0.06 | -0.16 | 0.52* | -0.27 | -0.07 | 0.63* | -0.30 | -0.14 | -0.35 | 0.59* | 0.64* | 1.00 |

*Note:* * p < 0.001

*Table 5. NON-RELIGIOUS PATIENTS: the matrix of inter-correlations of value statements*

| **Value**  **statements** | **1** | **2** | **3** | **4** | **5** | **6** | **7** | **8** | **9** | **10** | **11** | **12** | **13** | **14** | **15** | **16** |
| --- | --- | --- | --- | --- | --- | --- | --- | --- | --- | --- | --- | --- | --- | --- | --- | --- |
| **1** | 1.00 |  |  |  |  |  |  |  |  |  |  |  |  |  |  |  |
| **2** | 0.29 | 1.00 |  |  |  |  |  |  |  |  |  |  |  |  |  |  |
| **3** | 0.50* | 0.43 | 1.00 |  |  |  |  |  |  |  |  |  |  |  |  |  |
| **4** | 0.03 | 0.28 | 0.18 | 1.00 |  |  |  |  |  |  |  |  |  |  |  |  |
| **5** | 0.35 | 0.39 | 0.34 | 0.02 | 1.00 |  |  |  |  |  |  |  |  |  |  |  |
| **6** | 0.28 | 0.32 | 0.20 | 0.21 | 0.50* | 1.00 |  |  |  |  |  |  |  |  |  |  |
| **7** | 0.38 | 0.08 | 0.18 | 0.08 | 0.18 | 0.15 | 1.00 |  |  |  |  |  |  |  |  |  |
| **8** | 0.10 | 0.36 | 0.19 | 0.35 | 0.36 | 0.55* | -0.13 | 1.00 |  |  |  |  |  |  |  |  |
| **9** | 0.06 | 0.30 | 0.31 | 0.32 | 0.04 | 0.36 | -0.06 | 0.45 | 1.00 |  |  |  |  |  |  |  |
| **10** | 0.35 | 0.27 | 0.17 | 0.17 | 0.30 | 0.14 | 0.52* | 0.11 | 0.08 | 1.00 |  |  |  |  |  |  |
| **11** | -0.14 | 0.10 | 0.06 | 0.32 | 0.24 | 0.26 | -0.22 | 0.46 | 0.15 | -0.00 | 1.00 |  |  |  |  |  |
| **12** | -0.08 | 0.14 | 0.06 | 0.45 | 0.04 | 0.24 | -0.15 | 0.31 | 0.49 | 0.13 | 0.28 | 1.00 |  |  |  |  |
| **13** | -0.29 | 0.13 | -0.17 | 0.44 | 0.07 | 0.38 | -0.13 | 0.53* | 0.30 | 0.03 | 0.54* | 0.54* | 1.00 |  |  |  |
| **14** | 0.32 | 0.20 | -0.03 | 0.19 | 0.05 | 0.14 | 0.58* | -0.00 | 0.05 | 0.59* | -0.03 | 0.07 | 0.08 | 1.00 |  |  |
| **15** | 0.57* | 0.20 | 0.29 | 0.07 | 0.21 | 0.10 | 0.50* | -0.17 | -0.09 | 0.37 | -0.24 | -0.16 | -0.34 | 0.42 | 1.00 |  |
| **16** | 0.30 | 0.24 | 0.40 | 0.12 | 0.18 | -0.09 | 0.10 | -0.05 | -0.05 | 0.15 | 0.06 | -0.19 | -0.27 | -0.10 | 0.31 | 1.00 |

*Note:* * p < 0.001

*Table 6. HEALTHY NON-BELIEVERS: the matrix of inter-correlations of value statements*

| **Value**  **statements** | **1** | **2** | **3** | **4** | **5** | **6** | **7** | **8** | **9** | **10** |  | **12** | **13** | **14** | **15** | **16** |
| --- | --- | --- | --- | --- | --- | --- | --- | --- | --- | --- | --- | --- | --- | --- | --- | --- |
| **1** | 1.00 |  |  |  |  |  |  |  |  |  |  |  |  |  |  |  |
| **2** | 0.75* | 1.00 |  |  |  |  |  |  |  |  |  |  |  |  |  |  |
| **3** | 0.67* | 0.80* | 1.00 |  |  |  |  |  |  |  |  |  |  |  |  |  |
| **4** | 0.56* | 0.69* | 0.68* | 1.00 |  |  |  |  |  |  |  |  |  |  |  |  |
| **5** | 0.49 | 0.66* | 0.57* | 0.59* | 1.00 |  |  |  |  |  |  |  |  |  |  |  |
| **6** | 0.35 | 0.45 | 0.47 | 0.40 | 0.55* | 1.00 |  |  |  |  |  |  |  |  |  |  |
| **7** | 0.61* | 0.62* | 0.52* | 0.52* | 0.46 | 0.41 | 1.00 |  |  |  |  |  |  |  |  |  |
| **8** | 0.40 | 0.53* | 0.62* | 0.53* | 0.62* | 0.55* | 0.45 | 1.00 |  |  |  |  |  |  |  |  |
| **9** | 0.49 | 0.67* | 0.66* | 0.77* | 0.67* | 0.50* | 0.51* | 0.61* | 1.00 |  |  |  |  |  |  |  |
| **10** | 0.65* | 0.82* | 0.73* | 0.61* | 0.67* | 0.53* | 0.68* | 0.62* | 0.61* | 1.00 |  |  |  |  |  |  |
| **11** | 0.01 | 0.05 | 0.11 | 0.26 | 0.29 | 0.57* | 0.16 | 0.34 | 0.26 | 0.25 | 1.00 |  |  |  |  |  |
| **12** | 0.14 | 0.30 | 0.34 | 0.48 | 0.51* | 0.46 | 0.28 | 0.52* | 0.60* | 0.36 | 0.47 | 1.00 |  |  |  |  |
| **13** | 0.07 | 0.07 | 0.12 | 0.28 | 0.41 | 0.46 | 0.27 | 0.45 | 0.33 | 0.26 | 0.69* | 0.61* | 1.00 |  |  |  |
| **14** | 0.59* | 0.52* | 0.48 | 0.57* | 0.46 | 0.42 | 0.56* | 0.36 | 0.52* | 0.56* | 0.23 | 0.37 | 0.25 | 1.00 |  |  |
| **15** | 0.80* | 0.76* | 0.69* | 0.65* | 0.46 | 0.34 | 0.63* | 0.37 | 0.55* | 0.74* | 0.08 | 0.21 | 0.08 | 0.65* | 1.00 |  |
| **16** | 0.58* | 0.73* | 0.66* | 0.41 | 0.51* | 0.22 | 0.39 | 0.36 | 0.39 | 0.68* | -0.09 | 0.10 | -0.06 | 0.27 | 0.59* | 1.00 |

*Note:* * p < 0.001
